# Probe‐based confocal laser endomicroscopy for rapid on‐site evaluation of transbronchial biopsy specimens

**DOI:** 10.1111/1759-7714.13089

**Published:** 2019-05-06

**Authors:** Masao Takemura, Noriaki Kurimoto, Masahiro Hoshikawa, Toshitaka Maeno, Takeshi Hisada, Masahiko Kurabayashi, Takeo Inoue, Teruomi Miyazawa, Masamichi Mineshita

**Affiliations:** ^1^ Division of Respiratory Medicine, Department of Internal Medicine St. Marianna University School of Medicine Kawasaki Japan; ^2^ Department of Respiratory Medicine Gunma University Graduate School of Medicine Maebashi Japan; ^3^ Division of Medical Oncology and Respiratory Medicine, Department of Internal Medicine Shimane University Hospital Izumo Japan; ^4^ Department of Pathology St. Marianna University School of Medicine Kawasaki Japan; ^5^ Department of Cardiovascular Medicine Gunma University Graduate School of Medicine Maebashi Japan

**Keywords:** Acriflavine, bronchoscope, lung cancer

## Abstract

**Background:**

Probe‐based confocal laser endomicroscopy (pCLE) is a novel, noninvasive technology that provides real‐time lung imaging during bronchoscopy. pCLE shows the elastic fiber network without the use of a fluorescent dye. Elastic fibers produce argon laser‐induced autofluorescence at a wavelength of 488 nm, but tumor cells do not produce autofluorescence at this wavelength. As a result, the tumor cells cannot be observed directly. Therefore, we stained transbronchial biopsy (TBB) specimens with acriflavine to evaluate the benign and malignant structures using pCLE of ex vivo samples and to determine whether rapid histopathological diagnosis of TBB specimens could be made via pCLE.

**Methods:**

After bronchoscopy, 36 TBB specimens were stained with acriflavine and observed using pCLE. Benign and malignant lesions were classified by cell density and nuclear magnitude disparity.

**Results:**

We defined the confocal laser endomicroscopic atypia classification from the findings of the cells. The sensitivity for malignancy was 91.3%, and the specificity was 76.9%. Both inter‐observer (κ = 0.48) and intra‐observer (κ = 0.57) agreement confirmed moderate agreement.

**Conclusion:**

pCLE with acriflavine staining was useful to differentiate malignant from benign TBB specimens, and might be useful as a substitute for rapid on‐site evaluation of histopathological diagnosis.

## Introduction

Probe‐based confocal laser endomicroscopy (pCLE) is a novel technology for real‐time imaging during bronchoscopy that uses an argon laser with a wavelength of 488 nm to show the elastic fiber network and airway epithelium without the use of fluorescent dyes.

The principle of the confocal optical system was developed by Minsky in 1955,^1^ and it has been used in confocal laser microscopy since the 1980s. Since 2000, confocal optical technology has been applied to endoscopy, and its usefulness in gastrointestinal endoscopy has been investigated.^2^, ^3^, ^4^ In the field of bronchoscopy, Thibervill *et al.* reported pCLE imaging of the alveolus in 2009, and various reports of this imaging technique have subsequently been published.^5^ Specifically, several reports have mentioned that pCLE can visualize the degeneration of elastic fibers (fragmentation, disorganization, clumping, and thickening) and shows dark hollow patterns with no elastic fiber structure as a result of destruction by tumor growth.^6^, ^7^ These findings correspond to interstitial changes that occur around cancer cells. Fuchs *et al.* stained cellular nuclei using acriflavine and reported features of normal mucosa, inflammation/regeneration, and neoplastic lesions from characteristic structures of the cytoplasm and nucleus of airway epithelial cells and tissue architecture on pCLE analysis.^8^

Rapid on‐site evaluation (ROSE) has been established as a method of cytological diagnosis that can be performed during bronchoscopy. The use of ROSE is expected to improve diagnostic rates, shorten examination time, and reduce cost.^9^, ^10^, ^11^, ^12^ However, to date, no method has been established for the rapid on‐site histopathological diagnosis of TBB specimens in the bronchoscopy suite. pCLE is a very fast image processing system that provides real‐time evaluation of tissues and therefore, may be a tool that can be used during bronchoscopy for rapid on‐site histopathological diagnosis.

As no reports of the cellular findings with acriflavine staining in peripheral lung tissue have been published, we evaluated benign and malignant structures using pCLE with acriflavine staining of ex vivo samples and determined whether rapid on‐site histopathological diagnosis of transbronchial biopsy (TBB) specimens is possible with pCLE.

## Methods

### Study design

This prospective, observational ex vivo study was conducted with the approval of the Institutional Review Board of St. Marianna University Hospital (No. 2676). From April 2015 to January 2016, 38 patients underwent bronchoscopy at St. Marianna University, and TBB specimens were obtained from all patients. In two cases, the specimens were too small and could not be evaluated by pCLE, thus a final sample of 36 TBB specimens were examined via pCLE. Informed consent was obtained from all patients.

### Probe‐based confocal laser endomicroscopy (pCLE)

pCLE was performed using the Cellvizio system (Mauna Kea Technologies, Paris, France) set to a wavelength of 488 nm. A 1.4 mm diameter miniprobe (DEMO‐probe; Mauna Kea Technologies) was used. The probe has a lateral resolution of 3.5 μm, a field of view of 600 × 500 μm^2^, and a penetration depth of 0–50 μm.

### Bronchoscopic procedures

Pulse oximetry was performed during bronchoscopy, and blood pressure was measured every five minutes. Oxygen was supplied by a nasal cannula, and the flow was adjusted upward (as needed) from 2 L/minutes to continuously maintain pulse oximetry saturation above 90%. Conscious sedation was administered intravenously during all procedures. Evaluation of the central airways was performed using a flexible bronchoscope (BF‐P260F; Olympus, Tokyo, Japan), which was then advanced into the most distally accessible bronchi. The bronchoscope has a 4 mm external diameter and a 2 mm working channel. A 20 MHz radial‐type ultrasound probe (UM‐S20‐17S, Olympus) covered by a guide sheath (Guide Sheath Kit, K‐201, Olympus) was introduced into the lesion via the working channel of the bronchoscope. The probe was withdrawn, while the guide sheath was left in situ. Brush or biopsy forceps were introduced through the guide sheath into the lesion. On average, brushing and biopsies were performed five times.

### Transbronchial biopsy (TBB) specimens

TBB specimens were stored in a 10% formalin solution to maintain the quality of the tissue for pathology and then removed and placed on gauze for observation via pCLE. TBB was performed approximately five times in each patient, and the pCLE procedure was observed in one subject. During observation, a syringe was used to apply approximately two drops of 0.025% acriflavine solution to each specimen via a 25‐gauge needle. Between 1–2 minutes after the application of the acriflavine solution, the upper and bottom surfaces of the TBB specimens were observed. The pCLE images were recorded for analysis at a later date.

### Pathology

After evaluation via pCLE, the specimens were embedded in paraffin blocks. The blocks were sliced into 3 μm thick sections and stained using standard hematoxylin and eosin (HE) stain. Subsequently, light microscopic analysis of the histological specimens was performed.

### Pattern classification of pCLE images

**Figure 1:**
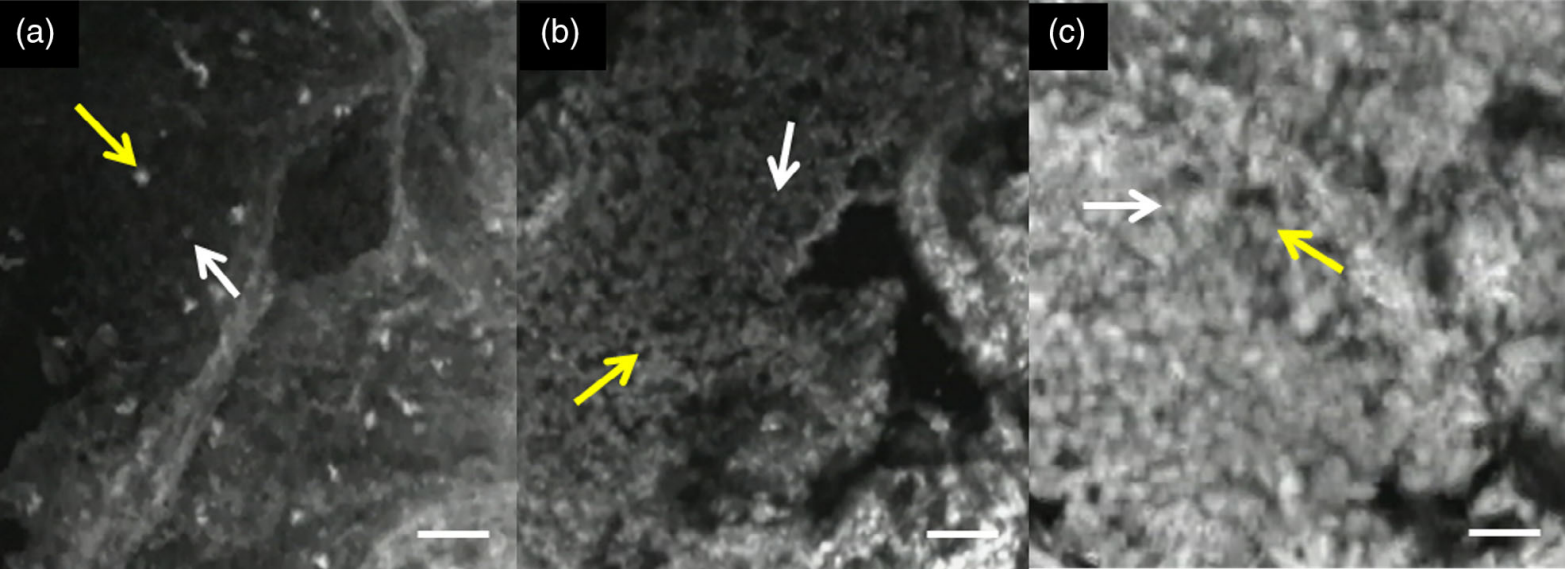
Confocal laser endomicroscopic atypia (CLEA) classification via probe‐based confocal laser endomicroscopy images. (**a**) CLEA −, small cell size is 7.5 μm (white arrow), large cell size is 11.3 μm (yellow arrow). (**b**) CLEA ±, small cell size is 4.1 μm (white arrow), large cell size is 6.3 μm (yellow arrow). (**c**) CLEA +, small cell size is 8.1 μm (white arrow), large cell size is 16.3 μm (yellow arrow). Scale bar = 50 μm.

Fuchs *et al.* analyzed confocal images of central airways stained with acriflavine.^8^ They identified criteria for determining the presence of normal mucosa and neoplastic or non‐neoplastic tissue based on the characteristic structure of the cytoplasm and nuclei of airway epithelial cells and tissue architecture via pCLE analysis. However, there are currently no criteria for identifying peripheral tissue via pCLE analysis. Therefore, we developed the confocal laser endomicroscopic atypia (CLEA) classification based on Fuchs's criteria and Minami's endocytoscopic atypia (ECA) classification used in the field of gastrointestinal endoscopy.^13^ We established the CLEA classifications as follows:

1. CLEA −: Cell density is low and nuclear size is uniform (Fig 1a).
2. CLEA ±: Cell density is high compared to the CLEA − type, or the enlarged nucleus is less than twice the short length of the small nucleus in one field of view (Fig 1b).
3. CLEA +: Cell density is high and the short length of the enlarged nucleus is greater than or equal to twice the short length of the small nucleus in one field of view (Fig 1c).

Because tumor cells have characteristics of high cell density and anisonucleosis, CLEA − or CLEA ± was defined as benign, while CLEA + was defined as malignant. The sensitivity and specificity of the CLEA classification for malignancy were then calculated.

The pCLE images were evaluated by two independent pulmonologists (observer A and B) proficient in bronchoscopy and experienced with pCLE. For inter‐observer reliability, observer A and B classified pCLE images, and for intra‐observer reliability, one observer (observer A) classified pCLE images within an interval of two months (observer A’).

As a method of evaluation, pCLE images were recorded in approximately 1–2 minutes of video. The images were classified into the three categories of the CLEA classification. Each evaluation was performed using the same video, with the observer blinded to the histopathological diagnosis. CLEA classification was conducted by comparing each image to Figure 1.

## Analysis

To confirm the validity of pCLE imaging, observer agreement was evaluated by analyzing inter‐observer and intra‐observer reliabilities.^14^ Cohen's κ values were then calculated to evaluate the degree of agreement, which was defined as: poor, κ < 0; slight, κ = 0–0.20; fair, κ = 0.21–0.40; moderate, κ = 0.41–0.60; substantial, κ = 0.61–0.80; and almost perfect, κ = 0.81–1.00.^14^ Sensitivity and specificity were calculated to evaluate the usefulness of diagnosis with pCLE. Using the 2 × 2 table, the following were calculated: sensitivity = true positive/(true positive + false negative) and specificity = true negative/(true negative + false positive).^15^

## Results

The histopathological diagnosis of the 36 TBB specimens was malignant in 23 cases (14 adenocarcinoma, 4 squamous cell carcinoma, 3 small cell carcinoma, 1 large cell neuroendocrine carcinoma, and 1 dermatofibrosarcoma) and benign in 13 cases (4 fibrosis, 3 inflammation, 1 amyloidosis, and 5 normal alveolus) (Table 1).

**Table 1:** Histopathological diagnoses of the 36 cases in this study

Pathology		N
Malignancy (*n* = 23)	Adenocarcinoma	14
	Squamous cell carcinoma	4
	Small cell carcinoma	3
	Large cell neuroendocrine carcinoma	1
	Dermatofibrosarcoma	1
Benign (*n* = 13)	Inflammation	3
	Fibrosis	4
	Amyloidosis	1
	Normal alveolus	5
	Total	36

Three representative cases are presented.

**Case 1 : Adenocarcinoma, CLEA +**

**Figure 2:**
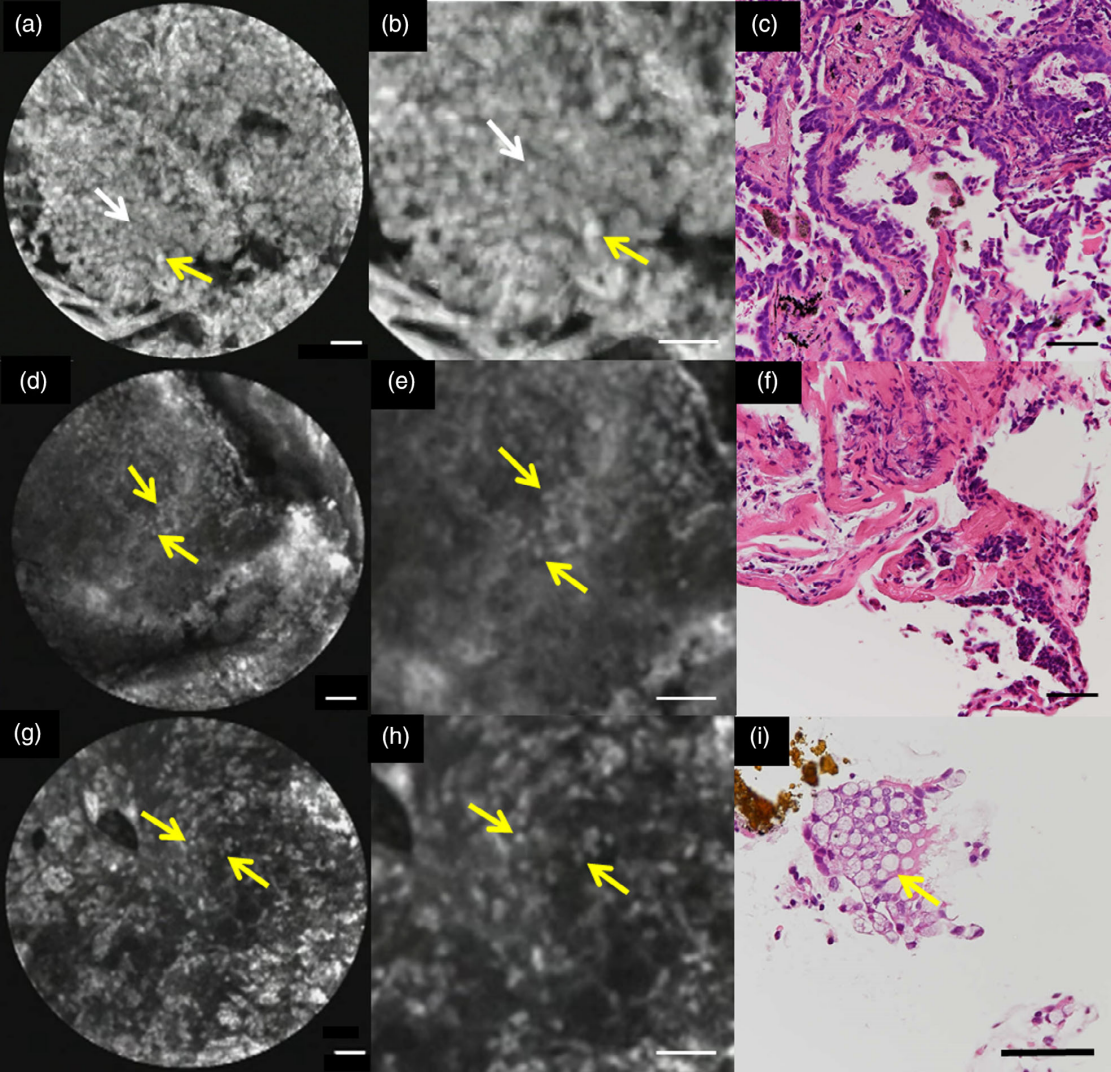
Representative case of the confocal laser endomicroscopic atypia (CLEA) classification. CLEA +: (**a**) After staining with acriflavine, a high density of cells and different sizes of nuclei are observed (white arrow, small cell; yellow arrow, large cell). (**b**) Four time magnification of (**a**) shows small (white arrow) and large (yellow arrow) cells. (**c**) Hematoxylin and eosin (HE) staining shows that the tumor cells proliferate in a papillary pattern. CLEA ±: (**d**) After staining with acriflavine, the density of cells is low compared to CLEA +, while the nuclear size is almost uniform (yellow arrow). (**e**) Four time magnification of (**d**) shows almost uniform nuclear size (yellow arrow). (**f**) HE staining shows atypical cells. CLEA –: (**g**) After staining with acriflavine, the density of cells is low, and the size of the nucleus is uniform (yellow arrow). (**h**) Four time magnification of (**g**) shows almost uniform nuclear size (yellow arrow). (**i**) HE staining shows only goblet cells (yellow arrow). Scale bar = 50 μm.

After staining with acriflavine, high‐density cells with various sized nuclei were visible, and the enlarged nucleus was greater than or equal to twice the short length of the small nucleus. After HE staining, the tumor cells proliferated in a papillary growth pattern. Nuclear fission was recognized, and nuclear sizes varied (Fig 2a–c).

**Case 2: Fibrosis, CLEA ±**

After staining with acriflavine, the cell density was low compared to the pCLE findings of CLEA + (Fig 2a), and the short length of the enlarged nucleus was less than twice the short length of the small nucleus. After HE staining, atypical cells were observed, but there were no findings to support the diagnosis of a malignant tumor (Fig 2d–f).

**Case 3: Normal cells, CLEA –**

After staining with acriflavine, the cell density was low, the sizes of the nuclei were uniform, and no elastic fibers were observed. HE staining only showed goblet cells (Fig 2g–i).

Tables 2 and 3 show the inter‐observer and intra‐observer agreement, respectively. Both inter‐observer (κ = 0.48) and intra‐observer (κ = 0.57) agreement was moderate. Based on this agreement, the diagnostic rate of observer A was subsequently evaluated. Table 4 shows the results of the diagnostic yield of pCLE for TBB specimens presented in a 2 × 2 table by observer A. The sensitivity and specificity using the CLEA classification for malignancy were 91.3% and 76.9%, respectively. The diagnostic accuracy of the CLEA classification for malignancy was 86.1%. There were 14 false‐positive or false‐negative cases, defined as at least one incorrect diagnosis among the three observations of observer A, B, and A’ (false‐positive cases 7, false‐negative cases 7). In the false‐positive cases, two inflammation, three fibrosis, one amyloidosis, and one normal alveolus case were misdiagnosed at least once during the three observations. Amyloidosis and one inflammation case were diagnosed as false positive by pCLE findings in all three observations. In the false‐negative cases, three adenocarcinoma, one squamous cell carcinoma, and three small cell carcinoma cases were misdiagnosed at least once during the three observations. In small cell carcinoma, all three cases were diagnosed as false negative in one of the three observations.

**Table 2:** Inter‐observer agreement

		CLEA (Observer A)			
		+ †	± ‡	– §	Total
CLEA (Observer B)	+	19	1	1	21
	±	2	6	1	9
	−	3	2	1	6
	Total	24	9	3	36
					κ = 0.48¶

† Confocal laser endomicroscopic atypia (CLEA) +: Cell density is high and the short length of the enlarged nucleus is greater than or equal to twice the short length of the small nucleus in one field of view.

‡ CLEA ±: Cell density is high compared to the CLEA − type, or the enlarged nucleus is less than twice the short length of the small nucleus in one field of view.

§ CLEA −: Cell density is low and nuclear size is uniform.

¶ κ refers to Cohen's κ values. κ = 0.41–0.60 has moderate agreement.

**Table 3:** Intra‐observer agreement

		CLEA (Observer A)			
		+ †	± ‡	– §	Total
CLEA (Observer A’) ¶	+	22	5	0	27
	±	2	4	0	6
	−	0	0	3	3
	Total	24	9	3	36
					κ = 0.57††

† Confocal laser endomicroscopic atypia (CLEA) +: Cell density is high and the short length of the enlarged nucleus is greater than or equal to twice the short length of the small nucleus in one field of view.

‡ CLEA ±: Cell density is high compared to the CLEA − type, or the enlarged nucleus is less than twice the short length of the small nucleus in one field of view.

§ CLEA −: Cell density is low and nuclear size is uniform.

¶ Observer A’ = Evaluation after two months from the first evaluation of observer A.

†† κ refers to Cohen's κ values. κ = 0.41–0.60 has moderate agreement.

**Table 4:** Diagnostic yield of pCLE for transbronchial biopsy specimens

		Pathology		
Method		Malignancy	No malignancy	Total
pCLE†	Malignancy	21	3	24
	No malignancy	2	10	12
	Total	23	13	36

† Sensitivity, 91.3%; specificity, 76.9%; and diagnostic accuracy, 86.1%. pCLE, probe‐based confocal laser endomicroscopy.

## Discussion

In the present study, the CLEA classification for pCLE imaging with acriflavine staining was found to be useful for diagnosing TBB specimens.

Using pCLE, elastic fibers were clearly visible in lung tissue. Because pCLE cannot show cells without any dye, Fuchs *et al.* used acriflavine to observe tumor cells by spraying it into the central airway system.^8^ They classified the images into three different types including normal mucosa, inflammation/regeneration, and neoplastic lesions, and detected malignant lesions with a sensitivity of 96.0% and specificity of 87.1%. Because no classifications have been established for peripheral lung lesions, we defined the CLEA classification based on cell density and nuclear size and evaluated the suitability of this classification for differentiating benign and malignant lesions. Our results demonstrated that pCLE with acriflavine provided a sensitivity of 91.3% and a specificity of 76.9% for the diagnosis of malignant lesions.

In bronchoscopy, ROSE is commonly used for endobronchial ultrasound‐guided transbronchial needle aspiration and has improved the diagnostic rate and reduced examination time and cost.^9^, ^10^, ^11^, ^12^ However, in lung biopsy, there is currently no method to evaluate TBB specimens on site. Therefore, establishment of a ROSE method is required for the field of bronchoscopy. In the present study, a high diagnostic rate could be obtained by evaluating TBB specimens with pCLE. It was possible to classify by reviewing a 1–2 minute video, thus pCLE might be a useful tool for rapid on‐site histopathological diagnosis. The DEMO‐probe was used to evaluate TBB specimens. Although this probe cannot be used for observation in vivo, it can be repeatedly used for observation ex vivo. Therefore, it is possible to reduce the cost of each examination by using the probe repeatedly.

In the pCLE images, there were 14 cases of false‐positive or false‐negative evaluations by observers A, B, and A’. In the false‐positive cases in particular, one inflammatory case and one amyloidosis case did not match the diagnosis between the pathology and pCLE findings in all three observations. Inflammation caused the assembly of cells, and a slight difference in brightness of the stained cells was observed as a result of the three‐dimensional structure of the TBB specimen. Consequently, these cases were diagnosed as positive. Small cell carcinoma was observed in three cases. All three cases of small cell carcinoma were diagnosed as false negative in one of the three observations. We posit that the reason for these results is that the nucleolar size of small cell carcinoma is not as varied as adenocarcinoma and squamous cell carcinoma, and, therefore, the diagnosis was easily confused with inflammation.

Several agents, such as cresyl violet, methylene blue, toluidine blue, acriflavine, and fluorescein, have been used in previous studies to observe cells via pCLE in the fields of digestive endoscopy^16^ and oropharyngeal cancer.^17^ In the field of pulmonology, Fuchs *et al.* reported pCLE observations with fluorescein and acriflavine. Fluorescein was intravenously administered, and observation from the bronchial epithelium to the alveolus was commenced one minute after the administration of fluorescein. In the central airways, epithelial cells were not stained and could not be observed with fluorescein. In the alveoli, when pushing the probe toward the alveoli, bubbles developed in front of the tip, and the alveoli could not be observed.^18^ Acriflavine was applied directly to the bronchial epithelium, and pCLE showed the bronchial epithelial cells by staining with acriflavine. It showed good sensitivity and specificity for differentiating malignant from non‐malignant lesions.^8^ In addition, acriflavine was able to stain the nucleus in a short time, and hence, appears to be useful for rapid diagnosis. Therefore, we used acriflavine to observe cells in lung tissue via pCLE.

Acriflavine is a heteroaromatic dye with antibacterial and antiviral effects.^19^ It was originally developed as an antibiotic, and in the early years was used as a treatment for trypanosomiasis. Topically administrated, acriflavine passes cell membranes and displays a strong specificity for labeling acidic constituents. Acriflavine predominantly stains the nuclei of the surface of the epithelium. Endoscopy with acriflavine enables differentiation between columnar epithelial cells and goblet cells, and in various normal and pathologic patterns.^20^ As described above, in the field of digestive endoscopy, various types of cells can be confirmed with pCLE imaging. In bronchoscopy, the cell density and the size of the nuclei can be confirmed; however, the different types of cells cannot be confirmed.

In the present ex vivo study, 1–2 mm TBB specimens were investigated. Both pCLE images and pathological findings were observed in this small range, and comparison of pCLE images with pathological findings was relatively easy. However, pCLE images could evaluate the surface of TBB specimens sterically, and the observable depth was 0–50 μm while the slice of pathological specimens was from the center of the TBB specimen, and the thickness was approximately 3 μm. Therefore, the same area could not be identified in pCLE images and the pathological findings.

This study had several limitations. First, as described above, the cell type could not be distinguished in pCLE images, and the same area could not be identified in pCLE and pathological images. Second, minutes after applying acriflavine, the pCLE image stained by acriflavine changed. After approximately two minutes, we suspected that the pCLE images showed not only nuclei, but also cell cytoplasm, although this could not be definitively confirmed. Third, the specimens were fixed with formalin before pCLE observation. Formalin fixation might lead to degeneration of the cells, decreasing the quality of the images observed via pCLE. However, in the present study, because observation with pCLE and diagnosis by HE staining were performed using the same specimen, it was necessary to prevent degradation of the quality of the specimen used for HE staining. There was concern about breaking the tissue by strongly pressing the pCLE probe when observing the specimen without formalin fixation, thus the specimens were fixed with formalin immediately after biopsy. Therefore, it is possible that the quality of the image evaluated by pCLE was slightly inferior. Fourth, in order to perform uniform evaluation, it is necessary become accustomed to handling the probe. In the present study, as the same person handled the probe, the pCLE images were of a similar quality. However, evaluation of the pCLE images might change as a result of differences in the handling of the probe, and in the present study, an estimated five cases were required to make a similar evaluation.

Because the present study was an ex vivo study, acriflavine staining caused no adverse effects. There is no evidence demonstrating the safety of acriflavine for use in the human body. Obstoy *et al.* reported that acriflavine induced considerable cellular DNA damage with illumination at 488 nm for two minutes.^21^ Therefore, we should carefully consider whether acriflavine should be allowed for use in the in vivo staining of lung cells.

In conclusion, pCLE images with acriflavine staining were useful to differentiate between benign and malignant TBB specimens. In the future, pCLE might be used as a substitute for rapid on‐site histopathological diagnosis.

### Disclosure

No authors report any conflict of interest.
